# Intravenous immunoglobulins for the treatment of prolonged COVID-19 in immunocompromised patients: a brief report

**DOI:** 10.3389/fimmu.2024.1399180

**Published:** 2024-04-19

**Authors:** Bénédicte Billi, Paul Cholley, Vincent Grobost, Mélissa Clément, Virginie Rieu, Guillaume Le Guenno, Hervé Lobbes

**Affiliations:** ^1^ Service de Médecine Interne, Hôpital Estaing, Centre Hospitalier Universitaire de Clermont-Ferrand, Clermont-Ferrand, France; ^2^ Service de Radiologie, Hôpital Estaing, Centre Hospitalier Universitaire de Clermont-Ferrand, Clermont-Ferrand, France; ^3^ Service de Médecine Interne, Hôpital Henri Mondor, Aurillac, France; ^4^ Institut Pascal, Unité Mixte de Recherche (UMR) 6602, Centre National de la Recherche Scientifique, Université Clermont Auvergne, Clermont-Ferrand, France

**Keywords:** SARS-CoV-2, immunodeficiency, B-cell depletion, rituximab, intravenous immunoglobulin

## Abstract

Primary humoral deficiency and secondary B-cell depletion may lead to prolonged Sars-Cov-2 infection due to a decreased viral clearance. Prolonged infection is mainly driven by the lack of anti-Sars-Cov-2 immunoglobulin (IVIg) especially in patients with no vaccine response. Anti-spike immunoglobulin can be provided by infusion of convalescent patients’ plasma: recent studies highlighted that commercial immunoglobulin show high titers of neutralizing IgG. We conducted a single center retrospective cohort. We included 9 patients (6 males, median age 74 years old): one patient with X-linked agammaglobulinemia and 8 patients treated with rituximab (2 granulomatosis with polyangiitis, 1 neuromyelitis optica, 4 low grade B-cell lymphoma and 1 EBV post-transplant lymphoproliferative disorder). Mean serum globulin was 4 ± 1.6 g/L. 7/8 had received at least 3 doses of mRNA anti-Sars-Cov-2 vaccine (median 4) with no response (anti-Spike IgG 0 for 6 patients). In this specific population requiring oxygen therapy but no intensive care support, the administration of IVIg was well tolerated and provided a swift improvement of clinical status, a significant decrease of inflammation associated to the an improvement of radiological patterns. Our results suggest that immunoglobulin could be used as a salvage therapy as an alternative to convalescent plasma but highly stringent patient selection is required due to the worldwide shortage of IVIg.

## 1 Introduction

In patients with humoral deficiency, Covid-19 infection may be prolonged by a lack of viral clearance (1). It has been showed that the persistence of Sars-Cov-2 infection facilitates rapid viral evolution in immunocompromised patients (2). The usual care is based on the use of antiviral therapies or convalescent patients’ plasma carrying anti-Sars-Cov-2 antibodies (3). In a recent meta-analysis the use of convalescent plasma was associated with a significant decrease of mortality (4). The use of remdesivir as monotherapy or in combination with convalescent plasma (5) has been suggested for B cell-depleted patients, mostly in hematological malignancies. However, studies suggest that the use of remdesivir may lead to the emergence of multiresistant viral clones (6, 7).

Convalescent plasma is collected from patients who have recovered from Sars-Cov-2 infection (8). Convalescent plasma is polyclonal: the selection of plasma donor’s to provide a high load of neutralizing antibodies can be made on the titers of IgG and IgA (9) and some studies showed that plasma from vaccinated individuals provide a highly effective in vitro antiviral effect (10). Standard preparations of commercial IVIg contain > 98% of IgG: IgA and IgM are virtually absent whereas normal plasma contains about 72% of IgG, 17% of IgA and 10% of IgM (11). IVIg pre-pandemic commercial batches of IVIg was showed to contain cross-reactive Sars-Cov-2 receptor binding domain antibodies (12): as such, early clinical study suggested a potential benefit of IVIg for patients with severe COVID-19 (13). The spread of the disease lead to a progressive significant increase of Sars-Cov-2 antibodies in commercial IVIg batches (14–17) which may represent an alternative strategy to plasma (18) in immunocompromised patients.

## 2 Methods

We conducted a retrospective monocentric study in the Internal Medicine department of Clermont-Ferrand Estaing University Hospital from 01/01/2021 to 15/12/2023. The study was conducted in accordance with the Helsinki declaration and approved by local the Ethics Committee (International Review Board 00013412, “CHU de Clermont Ferrand IRB #1”, IRB number 2023-CF240) with compliance to the French policy of individual data protection. Inclusion criteria were adult immunocompromised patients characterized by: (i) primary or secondary B-cell depletion associated with reduction in serum immunoglobulin G (< 5 g/L), (ii) poor vaccine response or refusal of vaccination. Sars-Cov-2 infection was detected by PCR on nasopharyngeal swabs or by bronchoalveolar lavage.

## 3 Results

### 3.1 Baseline characteristics

**Table 1** summarizes the characteristics of the population. We included 9 patients (6 males), median age 74 years old (min 20, max 86). 8/9 had secondary immunodeficiency associated with rituximab therapy: 3 patients were treated for autoimmune diseases (2 for granulomatosis with polyangiitis, 1 for neuromyelitis optica), 4 for low grade B-cell lymphoma, and 1 patient received various immunosuppressive drugs for pulmonary transplantation and rituximab for EBV associated Post-transplant lymphoproliferative disorder. The last patient (#9) was treated with low dose intravenous immunoglobulin for X-linked agammaglobulinemia (CD19 0/mm3).

**Table 1:** Characteristics of adult immunocompromised patients with B-cell depletion and Sars-Cov2 infection treated by intravenous immunoglobulins.

Patient	1	2	3	4	5	6	7	8	9
Age (years)	42	20	85	86	82	82	72	74	42
Sex	female	female	male	male	male	male	male	female	male
Medical history	Pulmonary transplant (cystic fibrosis) HBP, PE, COPD, DM	none	none	HBP	HBP, cardiac failure, PE, DM	HBP, DM, CKD	none	HBP, DM	none
Underlying disease	PTLD	GPA	MZL	FL	GPA	CV, SS, MALT lymphoma	FL	NMO	x-linked agammaglobulinemia
Immunosuppressants	RTX, CS, tacrolimus, everolimus	RTX, CS	RTX, revlimid	RTX	RTX	RTX, CS	Obinutuzumab	RTX	*IVIg substitution*
Number of anti-covid vaccine doses	4	0	4	4	7	2	2	5	4
Previous treatment for COVID-19	None	tixagevimab-cilgavimab	ritonavir-nirmatrelvir	CS	CS	None	tixagevimab-cilgavimab	ritonavir-nirmatrelvir	None
Disease course duration before IVIg (weeks)	3.5	3.5	6.5	2	2.5	1	16	6	3
CRP at baseline (mg/L)	104	25	92	128	79	18	46	157	4
Total globulins at baseline(g/L)	4	4	2.3	2.8	4.9	6.8	5	1.9	8.9
Total globulins after IVIg (g/L)	6.9	6.2	6	5.4	NA	NA	NA	4.6	NA
Anti-spike antibody at baseline (BAU/mL)	8	0	0	0	0	0	NA	0	1360
Anti-spike antibody after IVIg (BAU/mL)	2309	5577	NA	NA	747	4474	504	333	NA
O2 (l/min)	2	0	0	8	2	3	0	3	0
Affected lung parenchyma at baseline (%)	39.5	20	23.9	40.7	18.3	NA	19.4	46	15.5
Affected lung parenchyma after IVg (%)	9.4	16	12.8	50.8	10.7	NA	8.6	41.2	9.8

COPD, chronic obstructive pulmonary disease; CKD, chronic kidney disease; CV, cryoglobulinemic vasculitis; CRP, C reactive protein; CS, corticosteroids; DM, diabetes mellitus; FL, follicular lymphoma; GPA, granulomatosis with polyangeitis; HBP, high blood pressure; MALT, mucosal associated lymphoid tissue; MZL, marginal zone lymphoma; NMO, neuromyelitis optica; PE, pulmonary embolism; PTLD, Post-transplant lymphoproliferative disease; RTX, rituximab; SS, Sjögren’s syndrome.

NA, Not Available.

Among the 8 patients with secondary immunodeficiency, mean baseline serum globulin was 4 ± 1.6 g/L. 7/8 had received at least 3 doses of mRNA anti-Sars-Cov-2 vaccine (median 4) with no response (anti-Spike IgG 0 for 6 patients). All strains were Omicron: the sub-variants were identified for 7/9 patients: 4 patients had BA.5 (22B) Omicron and 3 patients had XBB (22F) Omicron. For 2 patients, the sub-variants was not identifiable because of a too low viral load on the sample.

### 3.2 Treatment and clinical outcome.

All the patients were febrile, 55% required oxygen therapy. No patients required high flow oxygen or transfer to an intensive care unit. 66% received a treatment for COVID-19 prior to IVIg including nirmatrelvir-ritonavir (2/9, 22%), corticosteroids (2/9, 22%), and tixagevimab-cilgavimab (2/9, 22%). The IVIg infusion (CSL Behring Company, Privigen^®^) was administered 3.5 weeks (median) after positive Sars-Cov-2 PCR without any corticosteroids or antiviral treatment. 8 patients (including the patients treated for x-linked agammaglobulinemia) received a single dose of 1g/kg, one patient received a lower dosage (0.4 g/kg) because of chronic kidney disease.

Oxygen therapy was discontinued 4.5 days after IVIg infusion for each patient. The CRP significantly decreased 48 hours (34 ± 38 vs 72 mg ± 52 mg/L) and 7 to 10 days after IVIg injection (10 ± 12 mg/L, p < 0.001). Anti-spike IgG serology became positive for 6/8 patients (mean titer 2324 BAU, minimum 333 - maximum 5577). The delay between infection and IVIg initiation as well as previous treatment before IVIg for Sars-Cov-2 infection had no influence on the response to IVIg.

### 3.3 Radiological findings and pulmonary function test

**Figure 1:**
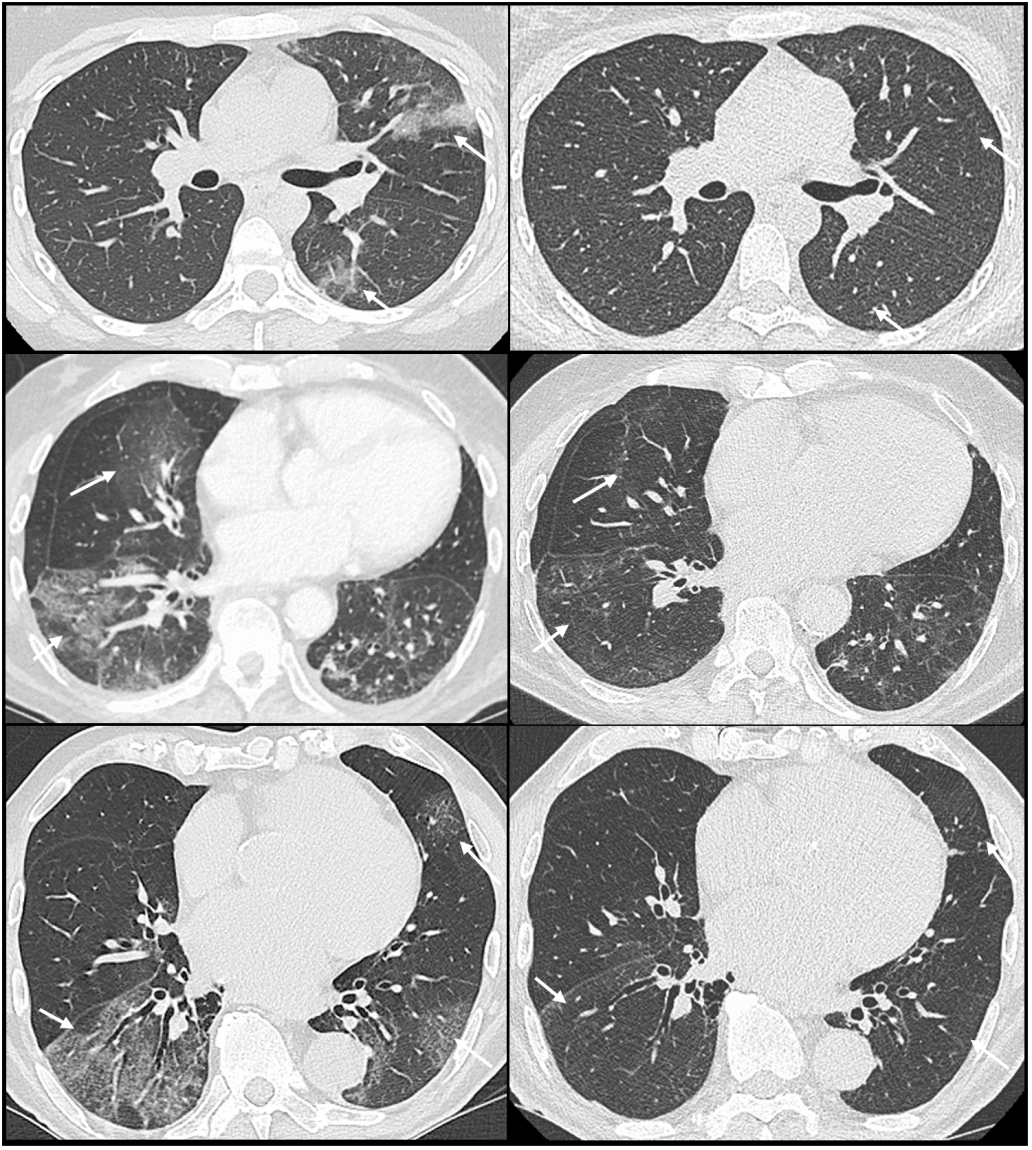
CT scan before (left) and after (right) intravenous immunoglobulin in immunocompromised patients with COVID-19 showing clear reduction in ground-glass opacities (arrows).

A systematic centralized evaluation of the CT scan before and after IVIg was performed, using automated lung segmentation and quantitative measurements (Thoracic VCAR^®^, GE Health Care Corporation). A significant improvement of the CT scan was found 1 to 2 months after infusion: the percentage of affected parenchyma decreased from 28 ± 12% to 20 ± 16% (p = 0.03) and the number of lobes decreased from 4.5 ± 0.2 to 3.4 ± 1 (p = 0.01). **Figure 1** shows representative changes of lung involvement before and after IVIg. Pulmonary function tests were available for 4 patients 3 months after IVIg: the forced vital capacity increased significantly for 2 patients (mean increase 425 mL (15%), p=0.02), while one patient remained stable (initially normal values) and one patient experienced alteration of her tests due to chronic graft rejection.

### 3.4 Clinical long-term outcomes

One patient discontinued rituximab treatment because of the stability of his follicular lymphoma. For the 7 remaining patients, the immunosuppressive therapy was not modified. One patient (#5) died 3 months after IVIg of a bacterial infection in a depressive geriatric background. Immediate IVIg tolerance was good for all the patients: no patients experienced adverse events.

At the last follow-up visit (median 5 months, minimum 3 - maximum 39 months), 3 patients were still receiving immunoglobulin substitution: patient #2 received subcutaneous immunoglobulin, whereas patient #7 and patient #9 received monthly IVIg.

Two patients (#2 and #3) experienced during the follow-up period a benign new Sars-Cov-2 infection (fever with no oxygen requirement), treated by ritonavir-nirmatrelvir. The other patients experienced no further sars-Cov-2 infection.

### 3.5 Comparison to patients treated by convalescent plasma therapy

During the same period, 6 immunocompromised patients (5 men, 1 woman; median age 63 years old) with no vaccine response (undetectable anti-spike antibodies despite 2 to 4 doses of vaccine) were treated by convalescent plasma therapy for COVID-19. Three patients received rituximab and various immunosuppressive drugs for autoimmune diseases (IgG4 related disease, warm autoimmune hemolytic anemia and polyangiitis microscopic) and 3 patients received rituximab (n=2) or obinutuzumab for low grade lymphoma (mantle cell lymphoma, follicular lymphoma and chronic lymphocytic leukemia). Only one patient required oxygen therapy before convalescent plasma infusion.

The **Table 2** shows the main features of the two groups. No difference were statistically significative in term of age, sex, BMI or comorbidity (high blood pressure or diabetes mellitus). Two patients treated with convalescent plasma experienced transient worsening after the infusion (fever, increase of CRP), and one patient required additional treatment with corticosteroids. All patients finally recovered and experienced a favorable long term outcome. The statistical analysis is hampered by the low size sample of both groups: we found a greater decrease of CRP 48 hours after IVIg than in plasma group but the difference did not reach statistical significance.

**Table 2:** Comparison of patients treated with IVIg or convalescent plasma for prolonged COVID-19.

	IVIg group (n=9)	Convalescent plasma group (n=6)	p
Age (years, mean ± SD)	65 ± 24	64.5 ± 14	0.9
Male sex (%)	67%	83%	0.6
BMI (kg.m^-2^, median [Q1;Q3])	22.7 ± 2	25.4 ± 3.8	0.1
Diabetes (%)	80%	60%	0.56
High blood pressure (%)	71%	75%	1
Number of anti-covid vaccine doses before treatment	3.5 ± 2	2 ± 1.7	0.14
Percentage of patients requiring oxygen	55%	17%	0.28
Total globulin before treatment	4 ± 1.6	3.5 ± 1.9	0.17
Affected lung parenchyma at baseline	28 ± 12	29 ± 17	0.87
Number of affected lung at baseline	4.5 ± 0.5	4.3 ± 0.8	0.65
CRP before treatment (mg/L)	73 ± 52	69 ± 46	0.89
CRP 48 hours after treatment (mg/L)	34.3 ± 38.4	37.1 ± 35.8	0.89
Decrease of CRP 48 hours after treatment (%)	60 ± 29	30 ± 44	0.12
CRP 7 to 10 days after treatment (mg/L)	10 ± 12	9 ± 5.3	0.87

## 4 Discussion

Early administration of IVIg in immunocompromised patients with B-cell depletion for the treatment of Sars-Cov-2 infection led to swift clinical, radiological and biological improvement. In our cohort, 6/9 patients had experienced the failure of a previous therapy for COVID-19 infection. A single dose of IVIg was used, without concomitant antiviral or cortisoteroid, with a good tolerance profile. Despite the lack of specific anti-Sars-Cov-2 antibodies, Xie et al. reported in 2020 a potential benefit of IVIg in severe COVID-19 (19), that may be due to the presence of cross-reactive Sars-Cov-2 receptor binding domain antibodies (12). In a retrospective case-control study in intensive care units, an increase of mortality was found in patients receiving IVIg (20) and in a multicenter retrospective study, no clear benefit of IVIg on hospitalization duration, mechanical ventilation or mortality rate was reported (21). A meta-analysis found similar results arguing against the use of IVIg (22): however, most studies of IVIg in COVID-19 have included critically-ill patients in intensive care units, most of them requiring mechanical ventilation.

Our population was very specific, as we included patients with B-cell immunodeficiency with severe impairment of globulin levels and no protective levels of IgG-anti-spike antibodies. Our results cannot be extended to severe patients admitted in intensive care units. The good tolerance profile and swift efficacy obtained suggest that IVIg could be used as a salvage therapy if convalescent plasma is not available. The transmission of IgG anti-Sars-COV2 has been clearly showed in X-linked agammaglobulinemic patients receiving IVIg (23), supporting the potential efficacy of IVIg administration as pre-exposure prophylaxis in immunocompromised patients especially as commercial batches contain increasing titers of neutralizing anti-Sars-Cov-2 IgG (24) but further studies are required to support the interest of this strategy. Nevertheless, the current shortage of IVIg dictates an extremely rigorous selection of patients eligible for this treatment.

## Data availability statement

The raw data supporting the conclusions of this article will be made available by the authors, without undue reservation.

## Ethics statement

The study was approved by local Ethics Committee (IRB00013412, “CHU de Clermont Ferrand IRB #1”, IRB number 2023-CF240) with compliance to the French policy of individual data protection.

## Author contributions

BB: Formal analysis, Investigation, Writing – original draft. PC: Formal analysis, Investigation, Writing – original draft. VG: Conceptualization, Formal analysis, Writing – review & editing. MC: Conceptualization, Formal analysis, Writing – review & editing. VR: Conceptualization, Formal analysis, Writing – review & editing. GL: Conceptualization, Formal analysis, Methodology, Writing – review & editing. HL: Conceptualization, Investigation, Project administration, Visualization, Writing – original draft, Writing – review & editing.
